# Male Stress Urinary Incontinence: A Review of Surgical Treatment Options and Outcomes

**DOI:** 10.1155/2012/287489

**Published:** 2012-05-08

**Authors:** Landon Trost, Daniel S. Elliott

**Affiliations:** Department of Urology, Mayo Clinic, Rochester, MN 55905, USA

## Abstract

*Introduction and Objective*. Iatrogenic male stress urinary incontinence (SUI) affects a percentage of men undergoing urologic procedures with a significant impact on quality of life. The treatment of male SUI has evolved significantly with multiple current options for treatment available. The current paper discusses preoperative evaluation of male SUI, available surgical options with reported outcomes, and postoperative complication management. *Methods*. A pubMed review of available literature was performed and summarized on articles reporting outcomes of placement of the artificial urinary sphincter (AUS) or male slings including the bone anchored sling (BAS), retrourethral transobturator sling (RTS), adjustable retropubic sling (ARS), and quadratic sling. *Results*. Reported rates of success (variably defined) for BAS, RTS, ARS, and AUS are 36–67%, 9–79%, 13–100%, and 59–91% respectively. Complications reported include infection, erosion, retention, explantation, and transient pain. Male slings are more commonly performed in cases of low-to-moderate SUI with decreasing success with higher degrees of preoperative incontinence. *Conclusions*. An increasing number of options continue to be developed for the management of male SUI. While the AUS remains the gold-standard therapy for SUI, male sling placement is a proven viable alternative therapy for low-to-moderate SUI.

## 1. Introduction

Urinary incontinence is estimated to affect 12–17% of US males, with increasing prevalence associated with aging [1, 2]. Stress urinary incontinence (SUI) as a subtype has been defined by the International Continence Society as the complaint of involuntary leakage on effort or exertion, or on sneezing or coughing [3]. Although any surgical or radiotherapeutic manipulation of the external urinary sphincter may result in SUI, radical prostatectomy (RP), transurethral resection of the prostate (TURP), and radiation therapy are most commonly associated with RP accounting for the majority of iatrogenic etiologies. The true prevalence of SUI following RP is unknown with widely varying estimates reported from 2 to 43%, which is likely reflective of differing surgical techniques, methodology, definitions, and followup performed, among others [4–9]. External beam radiation therapy and TURP are less commonly associated with SUI, with reported outcomes ranging from 1 to 16% and 1 to 3%, respectively [10–12]. Given that prostate cancer is the most commonly diagnosed malignancy in US males, the true scope and impact of iatrogenic male SUI on quality of life (QOL) is likely significant.

The treatment of male SUI has continued to improve since the introduction of the first externally worn urethral cuff by Foley in 1949. Subsequent modifications of an internally placed prosthesis by Kaufman in 1973 (Kaufmann III) and an internal reservoir by Rosen in 1976 led to the first completely internalized artificial urinary sphincter (AUS). Despite initial enthusiasm with the procedure, significant complications arose including urethral erosions and fistulae secondary to the continuously elevated urethral occlusion pressures. Subsequent advancements by American Medical Systems (AMS, Minnetonka, Minnesota) with the AS 721 led to reported success rates of 79% among 34 patients treated by Scott and colleagues [13]. Further improvements were later introduced including automatic cuff closure, cuff deactivation, and modifications to the narrow-backed cuff, all of which have continued to improve outcomes while decreasing complication rates. Currently, the most commonly utilized AUS is the AMS 800 (AMS, Minnetonka, Minnesota) which consists of a pump, reservoir, and urinary cuff. Since its popularization, the AUS has remained the gold-standard treatment for male SUI. 

Beginning in the late 1990s, the male sling was introduced as a surgical alternative to the AUS for patients with low volume incontinence (1–3 pads). Among other factors, one notable difference with male slings compared to the AUS is the lack of mechanical components, which reduces the potential for device failure. Although several variations on sling design exist, the most commonly published series available report on three specific designs, the suburethral bone-anchored slings (BAS), retrourethral transobturator slings (RTS), and adjustable retropubic slings (ARS).

With an increasing number of options available for the treatment of male SUI, it is important for treating clinicians to be aware of available therapeutic options, comparative outcomes, and associated complications. The current paper is outlined to review the clinical evaluation of males presenting with SUI, discuss the male sling and AUS as treatment options, review reported outcomes on therapies, briefly discuss management of common postoperative complications, and highlight potential future perspectives.

## 2. Clinical Evaluation

Males presenting with stress urinary incontinence should undergo a complete history and physical examination to include reviewing the underlying etiology and duration of the incontinence, current and prior urinary symptoms, history of genitourinary pathology (ex, nephrolithiasis, urothelial carcinoma), urinary tract infections, the degree and subjective bother of incontinence, and a review of prior procedures including radiation. Additional quantitative measures which may be employed include obtaining pad weights and standardized QOL questionnaires [14–16]. Patients should further undergo a genitourinary examination and be assessed as to their physical and mental capacity to function a potential AUS device.

Further testing may be individualized based on results obtained during the history and physical examination. All patients should undergo a routine urinalysis and postvoid residual to rule out concurrent infection and any degree of urinary retention. Additional testing may be obtained as clinically indicated including imaging (ex, history of nephrolithiasis, urothelial carcinoma), urine cytology (ex, irritative voiding symptoms, history of urothelial carcinoma), and PSA. Urodynamic studies are not routinely performed and are predominantly reserved for cases of suspected elevated bladder pressures or indeterminate/multifactorial etiologies for incontinence [17]. It is the author's practice to perform a cystoscopy on all patients considering surgical treatments for SUI to rule out anatomic abnormalities (ex, stricture, bladder neck contracture). Additionally, this permits filling of the bladder with saline followed by direct observation of the degree of stress urinary incontinence experienced.

Surgical candidates desiring a male sling or artificial urinary sphincter should be 6–12 months out from the initial event resulting in SUI as this permits resolution of concomitant urinary symptoms and allows sufficient time for spontaneous recovery of continence. Additionally, patients should ideally have a normal bladder capacity and compliance, have isolated SUI without significant urgency, be free of intraurethral and/or intravesical pathology, have sufficient physical and mental capacity to function a potential device, and be able to maintain his activities of daily living without need for assistance. Although none of these factors would preclude surgical intervention, each should be weighed in the clinical decision so as to reduce potential future complications. Patients with prior histories of urothelial carcinoma, nephrolithiasis, urethral stricture disease, and bladder neck contractures, among others should demonstrate a sufficient period of disease stability prior to consideration of sling/AUS placement to reduce the risk of erosions resulting from repeated interventions.

## 3. Treatment Options

Although numerous treatment options for male SUI exist, including penile clamps, transurethral bulking agents, or catheters (condom or indwelling), the most commonly utilized surgical therapies performed include placement of a male sling or AUS.

### 3.1. Male Sling

Since its initial introduction, the male sling has become increasingly utilized in cases of low-to-moderate volume (1–3 pads/day) incontinence. Although several variations of the male sling are currently available, the three subtypes with the most reported series available include the BAS, RTS, and ARS.

**Figure 1:**
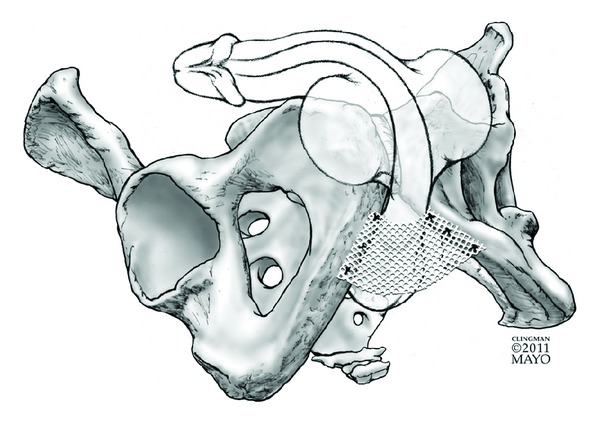
Graphical representation of the bone-anchored sling.

Bone-anchored slings result in compression to the bulbar urethra through placement of a synthetic or organic mesh which is secured to the inferior pubic ramus using six titanium screws. Sutures are subsequently secured to the screws and mesh material and tightened to result in appropriate tensioning. Following initial reports of degradation of organic materials, synthetic mesh (InVance; AMS, Minnetonka, Minnesota) has become the most commonly utilized material with the BAS [18]. See Figure 1 for graphical representation of BAS placement.

A second category of available male slings includes the RTS, with the AdVance (AMS, Minnetonka, Minnesota) and I-Stop TOMS (CL Medical, Lyon, France) slings most commonly employed. In contrast to the BAS which utilizes anchored sutures, the RTS is self-anchored with bilateral polypropylene mesh arms placed in a transobturator fashion. The sling portion is secured at the proximal bulbar urethra with continence achieved through subsequent elevation of the urethra.

**Figure 2:**
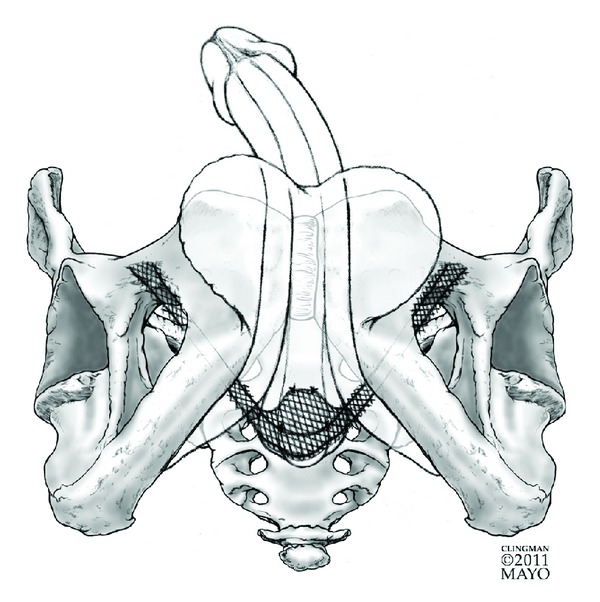
Graphical representation of the retrourethral transobturator sling.

Several reports have examined preoperative characteristics, surgical techniques, and postoperative management principles which have been associated with improved outcomes with RTS placement [29–31]. Preoperative characteristics found to be predictive of worsened outcomes include weakened residual sphincter function, incomplete sphincter closure, and lack of elongation of the coaptive sphincter zone. Intraoperative and postoperative factors associated with improved outcomes include tunneling of the sling arms into subcutaneous tissues to improve fixation, placing five or more stitches, using nonabsorbable stitches, and minimizing postoperative activity to reduce dislodgement. See Figure 2 for graphical representation of RTS placement.

**Figure 3:**
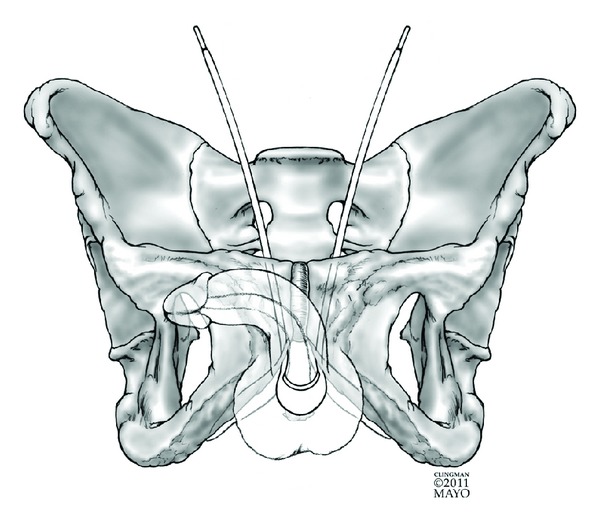
Graphical representation of the adjustable retropubic sling.

Similar to RTS, ARS (Reemex, Neomedic International, Terrasa, Spain; and Argus, Promedon SA, Cordoba, Argentina) are surgically placed at the proximal bulbar urethra, with traction sutures placed retropubically. The sutures are then tensioned at the level of the rectus fascia utilizing either a “veritensor” (Reemex) device or silicone columns and washers (Argus) to provide an appropriate level of urethral compression. See Figure 3 for graphical representation of ARS placement.

**Figure 4:**
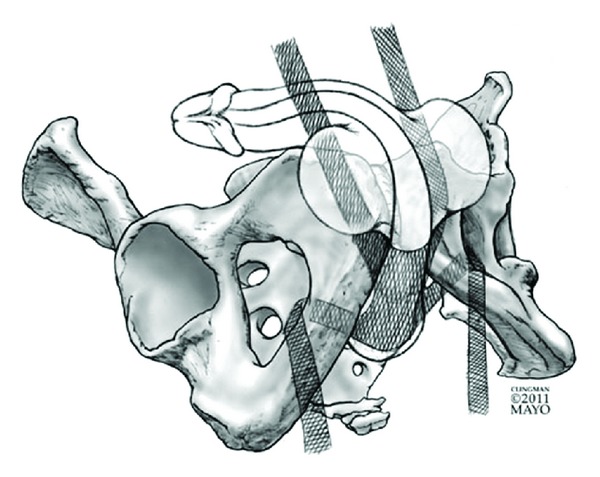
Graphical representation of the quadratic sling.

A fourth category of sling which has recently been introduced is the quadratic sling (Virtue, Coloplast, Humlebaek, Denmark). The sling consists of a broad-based mesh material placed over the bulbar urethra similar to the BAS. It is then self-secured with four mesh arms which are placed in both a transobturator (two arms) and prepubic (two arms) manner. The limbs may then be further secured to create additional points of fixation as needed. See Figure 4 for graphical representation of quadratic sling placement.

The hypothesized mechanism for improved continence with the various sling designs varies and is not thoroughly understood. Bone-anchored slings likely achieve direct compression of the bulbar urethra with subsequent increases in outflow resistance. In contrast, the mechanism for the RTS is based on the hypothesis that mild/moderate SUI results from compromise of periurethral supporting structures [32]. Through proximal placement of the mesh material, the dynamics of the bulbar urethra are modified to result in functional extension of the membranous and angulation of the bulbar urethra. The mechanisms for improved SUI with the ARS and quadratic sling are currently unknown and may result from a combination of urethral compression and angulation.

### 3.2. Artificial Urinary Sphincter

**Figure 5:**
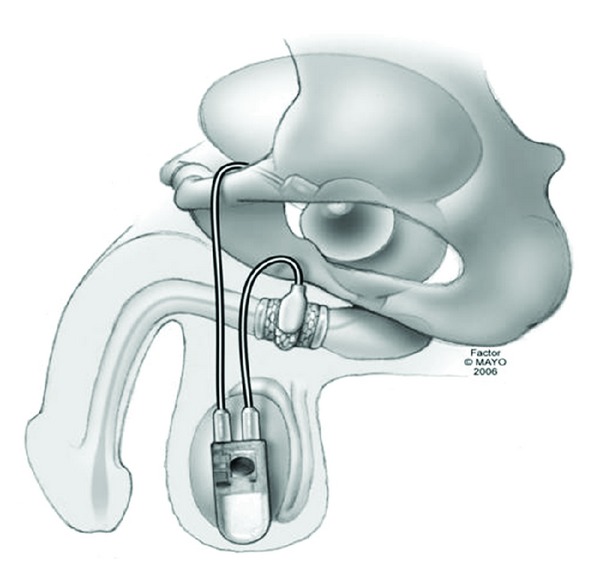
Graphical representation of the single-cuff artificial urinary sphincter.

Since its popularization in 1978, the AUS has arguably remained the gold-standard therapy for SUI. The currently available model, AMS 800 (AMS, Minnetonka, MN), consists of a pump, pressurized reservoir, and sphincter cuff. Cuff sizes range from 3.5 to 14.0 cm, and the reservoir is available in five pre-set pressures (41–50, 51–60, 61–70, 71–80, and 81–90 cm H_2_O) to adapt to various patient requirements. When cycled, the pump functions to actively shunt fluid from the cuff to the reservoir via a unidirectional valve, which additionally prevents uncontrolled retrograde transmission of fluid to the cuff. A refill-delay resistor maintains the cuff in an open state to permit voiding and subsequently permits transfer of fluid from the reservoir to the cuff. The pump also contains a deactivation button which permits the cuff to be placed in a locked “open” state when needed or to potentially reduce urethral atrophy [40]. See Figure 5 for graphical representation of AUS single cuff placement.

**Figure 6:**
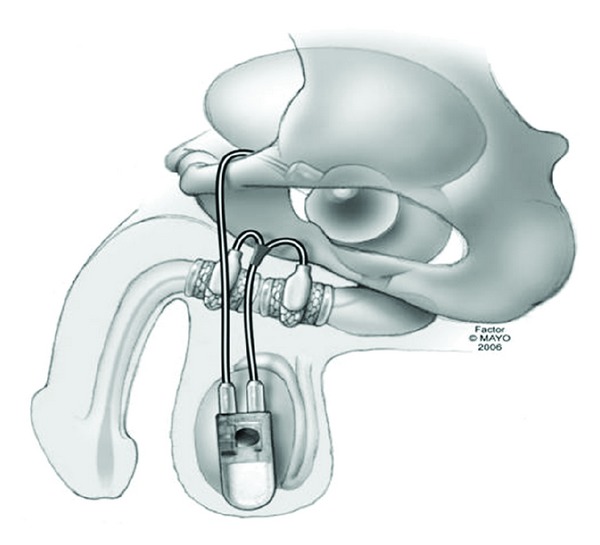
Graphical representation of the tandem-cuff artificial urinary sphincter.

Placement of the AUS is performed via a similar dissection to that of male sling insertion. The proximal bulbar urethra is identified, isolated, and measured, and an appropriately sized cuff is placed circumferentially. The reservoir is placed deep to the rectus sheath, and the pump is located in the inferior hemiscrotum. Contrast may be instilled in lieu of saline to permit future trouble shooting of the device, and the device is connected and cycled. Variations to placement of the AUS are frequently utilized in cases of recurrent incontinence following prior AUS placement, urethral atrophy, or prior device erosions/infections. In these settings, an AUS may be placed in “tandem” with an existing AUS and secured to the reservoir and pump with a Y-connect device [41]. See Figure 6 for graphical representation of AUS tandem cuff placement. Alternatively, a transcorporal dissection of the proximal bulbar urethral may be performed or alternative materials may be placed around the urethra including porcine small intestinal submucosa at the time of AUS cuff placement to provide additional tissue bulk [42–44]. 

In patients presenting with persistent incontinence following prior sling placement, an AUS may be placed, with dissection performed similar to a primary AUS procedure. In cases where the prior mesh is encountered, this may be incised without need for complete excision, and the cuff placed in the standard fashion.

## 4. Results

Multiple series are currently available reporting outcomes of the various male sling techniques and AUS implantation. However, given the nature of the studies performed and methodology for reporting, outcomes should be interpreted with caution. There is currently no accepted standard method for reporting pre- and postoperative degrees of incontinence or any consistent method for defining success with treatment. The majority of studies have poorly or undefined inclusion/exclusion criteria with significant heterogeneity of the patient population including inconsistent inclusion of patients with varied etiologies for SUI or prior radiation therapy. These factors, among others, limit the ability to draw comparisons between studies and techniques.

### 4.1. Male Sling

As the BAS has been available and utilized for a longer period of time than other slings, more studies are currently available for review with longer mean/median follow up periods. For the purposes of the current review, studies were included if they were published within the past 10 years and examined synthetic sling placement only, as organic sling material is no longer commonly employed. 

Overall results of the BAS demonstrated cure rates ranging from 37 to 67% with improvement noted in an additional 10–40% [19–28]. The wide range of results is likely secondary to surgical method, definitions for continence utilized and also may be due to a migration of case complexity. More recent reports have included an increased number of patients with prior radiation therapy and those with more severe preoperative incontinence. Several studies have noted significance in the association of preoperative continence and postoperative success rates with conflicting reports on the impact of radiation on overall success. Complications commonly reported include infection (2–15%), erosion (0–3%), de novo urgency/overactivity (0–14%), pain (0–73%) which typically resolves within 4 months, and sling removal (0–13%). See Table 1 for comparison of outcomes among patients undergoing BAS placement. 

Results from placement of the RTS have similarly demonstrated resolution or improvement in males with mild-to-moderate SUI in 9–62% and 16–46% of patients, respectively [33–39, 58]. With the notable exception of Cornel and colleagues who reported a success rate of 9% and failure rate of 46% among 35 patients, other studies report higher cure rates of 52–74% with improvements noted in and additional 16–27%. Complications reported with the RTS include temporary urinary retention <2 weeks (0–24%), urethral injury (0–3%), pain (0–34%), need for sling removal (0–4%), and dysuria (0–14%).

It is notable that four studies examining RTS were prospectively designed, with three accruing over 110 patients [33, 34, 38, 58]. As with the BAS groups, improved outcomes were noted among patients with decreased preoperative incontinence, with a trend towards increased failures noted among patients with preoperative radiation therapy [38].

Two studies of interest investigated the role for RTS as a salvage technique in cases of recurrent incontinence following prior anti-incontinence surgery. Christine and colleagues reviewed 19 patients undergoing RTS in patients with recurrent incontinence following prior AUS placement [35]. Patients had self-reported pre-op pad usages of 2–5 ppd. Following RTS placement, 15/19 (79%) reported requiring 0 ppd, with the remaining 4/19 (21%) describing improvement. Approximately half of the patients did not require reactivation of the sphincter.

Similarly, Soljanik and colleagues reported on 29 patients undergoing RTS following a previously failed sling procedure with preoperative mean pad requirement of 4.3 ppd. At 17 months followup, results demonstrated resolution of incontinence in 10/29 (35%) with improvement noted in an additional 16/29 (55%). These studies highlight the potential role for male sling placement as a potential adjunctive/salvage treatment; however, further validation is required prior to its consideration as a routine salvage measure. See Table 2 for comparison of outcomes among patients undergoing RTS placement.

A third category of currently available slings includes the ARS, with the Argus (Promedon SA, Cordoba, Argentina) and Reemex (Neomedic International, Terrasa, Spain) slings most commonly utilized. Results of initial and longer-term followup demonstrate success rates of 13–100% with larger series reporting rates of 54–79% [45, 47–52]. Patients required adjustments in 10–100% of cases, many of which required repeated anesthesia. Complication rates were noted to be significantly higher compared to other sling categories with infections (5–7%), erosion (3–13%), explantation (2–35%), bladder perforation (5–29%), retention (35%), and perineal pain (4–38%) most commonly reported. See Table 3 for comparison of outcomes among patients undergoing ARS placement.

One study of interest conducted by Tuygun and colleagues retrospectively compared the Argus (Promedon SA, Cordoba, Argentina) sling to the AMS 800 (AMS, Minnetonka, Minnesota) AUS [46]. A total of 16 patients with prior AUS erosions were treated with either ARS (*n* = 8) or repeat AUS (*n* = 8). Results demonstrated cure rates of 5/8 (63%) versus 1/8 (13) and improvement rates of 2/8 (25) versus 1/8 (13) for the AUS and sling, respectively.

A more recently released sling is the Virtue quadratic sling (Coloplast, Humlebaek, Denmark) with minimal data available on initial outcomes. The only currently published study was performed by Comiter and colleagues who reported initial outcomes of 22 patients undergoing sling placement with pre- and postoperative retrograde leak point pressures tested [59]. Results demonstrated improvements in the leak point pressure from preoperative 33 ± 9 to 69 ± 6 cm H_2_O. Although these results are an encouraging proof of concept, further data is currently pending.

### 4.2. Artificial Urinary Sphincter

Multiple series have reported on long-term AUS outcomes [53–57]. As the AUS has been available for use for a longer period of time than the male slings, mean follow-up periods are greater and range from 3 to 7.7 years. Similar to the reports on male slings, the definition for postoperative continence varies by study, with the most commonly utilized definition of 0-1 pads as being “continent.” Results demonstrate overall continence rates of 59–91% with the two studies which included over 100 patients reporting 69% and 82% success [54, 56]. Kim and colleagues reported on the long-term durability of the AUS and noted that patients with <4 years of follow-up experienced continence rates of 76% which increased to 89% in those with >8 years followup. This result appeared to correlate with the finding that the far majority of surgical revisions required were performed in the first 36–48 months with an overall, long-term mechanical failure rate of 36% noted at 10 years [54].

Similarly, Lai and colleagues reported on 270 patients with a mean followup of 3 years including some patients with >12 years followup [56]. Mean preoperative and postoperative pad use was 5.3 and 1.1 ppd, respectively. Thirty-four percent (60/176) of patients who presented with postprostatectomy incontinence (PPI) had undergone prior radiation therapy, with 34% and 43% of all PPI patients demonstrating detrusor instability and decreased bladder compliance on urodynamic studies, respectively. Patients who had previously undergone peri-urethral bulking agent administration or male sling placements did not demonstrate a decreased success rate compared to those who had not undergone similar procedures. Twenty-two percent (59/270) of patients ultimately required repeated surgical intervention secondary to complications at a median time of 14.4 months.

Common complications following AUS placement include urethral atrophy resulting in recurrent incontinence (4–10%), erosion (4–10%), infection (1–14%), and mechanical failure (0–29%). See Table 4 for comparison of outcomes among patients undergoing AUS placement.

Numerous additional studies have reported on salvage therapies available for prior AUS failures including tandem cuff placement, cuff downsizing, transcorporal cuff, and the use of biologic materials as a urethral bulking agent [41–43, 60–62]. Although a formal review of salvage therapies and outcomes is beyond the scope of the current review, one recent study with long-term followup examined primary single versus double cuff AUS placement and found no statistical difference in continence outcomes between treatment groups [55]. This would argue against the routine placement of tandem cuffs as a primary treatment modality. Similarly, the above procedures are predominantly reserved for use as a salvage technique rather than primary therapy.

Many studies have reported significant improvements in quality of life measures with both the male sling and AUS [28, 37, 39, 49, 63]. This likely highlights the significant impact that SUI has on overall quality of life as well as the improvements noted with its treatment. It is also important to note that with either male sling or AUS placement, it is uncommon to experience a complete resolution of incontinence with a return to preexisting baseline continence levels. As such, it is important to appropriately counsel patients as to reasonable postoperative expectations and potential complications.

## 5. Complications

Complications resulting from either male sling or AUS implantation may be categorized as occurring intraoperative, early postop (<90 days) or late postop (>90 days). Intraoperative complications may include urethral injury occurring at the time of urethral dissection or passage of a trocar for male sling placement. If a small injury is recognized, placement of the AUS/male sling may continue at a separate site to prevent subsequent erosions. A large urethral injury should be repaired primarily with the procedure aborted and a catheter placed. Bladder injuries occurring during trocar passage may be managed with repassing of the trocar and subsequent catheterization for a period of several days postoperatively. Given the relative incidence of bladder injury with retropubic sling placements, patients undergoing these procedures should undergo intraoperative cystoscopy to rule out bladder perforation.

Early postoperative complications include urinary retention, infection and/or erosion, perineal pain, and de novo detrusor overactivity. Urinary retention typically occurs secondary to postoperative edema and resolves spontaneously in the majority of cases. Persistent retention lasting >8 weeks may indicate inappropriate sizing of the sphincter cuff, overtensioning of the sling, or sling malposition. Retention is typically managed with in-and-out catheterization with suprapubic tube placement required in rare cases. In the case of AUS placement, it is the authors' preference to avoid indwelling catheters and to use a small (12 F) straight catheter when required in the postoperative period to reduce the risk of development of catheter-related erosions.

Infections of the AUS device or sling material may be secondary to unrecognized urethral erosion versus intraoperative contamination. Preoperative patient factors including repeated device placements, prior erosions, and radiation therapy all predispose patients towards a higher rate of postoperative infections. The most commonly isolated organisms with infection include *S. aureus, S. epidermidis*, Enterococcus, Methicillin resistant *S. aureus*, and gram-negative *bacilli* [64]. Infections occurring beyond 90 days may be related to hematogenous spread of bacteria at the time of additional procedures.

Urethral erosions occurring early in the postoperative period are likely secondary to unrecognized urethral injury occurring at the time of surgical implantation. Device erosions require explantation, even in the absence of infection, with possible repeat AUS/sling placement performed several months later pending sufficient recovery and absence of urethral stricture development.

Postoperative perineal pain is more common with male sling placement than AUS, with some authors noting pain in 100% of male sling patients for periods up to 4 months. Patients may additionally develop de novo detrusor overactivity, which may be managed with anticholinergic therapy as indicated.

Mechanical failure is unique to AUS devices and has been shown in one long-term followup of 100 patients undergoing AUS to have 5- and 10-year device failure-free rates of 74.8% and 70.1%, respectively [53]. Additional studies with follow-up periods >5 years confirm similar findings of device failure rates of 25–34% [54, 57, 65].

## 6. Patient Stratification

The decision as to which procedure to perform in males presenting with stress urinary incontinence is based on several factors. Most commonly, male slings are offered in cases of lower-volume incontinence (1–3 pads/day), or in the setting of complicating patient factors including inability to function the AUS pump. Placement of an AUS may be performed with any degree of SUI and may be employed in the setting of prior male sling failures.

There is currently no universally accepted standard by which patients are stratified into receiving a male sling versus AUS. Similarly, there are no currently accepted objective measures by which men are formally evaluated for stress incontinence. Evaluating clinicians may elect to stratify patients based on subjective reporting of pad usage, objectively obtained 24-hour pad weights, or by the degree of SUI visualized on examination. This lack of consensus on the clinical evaluation of males with SUI is mirrored in the available published literature which similarly lacks an accepted method of standard reporting.

Additionally, there are currently no publications which directly compare results for the various treatments of male SUI, and as such, it is not possible to directly compare reported outcomes between studies. Based on the reported literature available, it is not possible to definitively identify one sling procedure as superior over another.

In general, available data on the various male slings have shown a reduction in overall efficacy in patients with pre-surgical, higher volume incontinence (discussed further in Section 4), and therefore AUS is typically chosen in these cases. Alternatively, male slings may be preferred in cases of diminished hand and/or cognitive ability, regardless of degree of incontinence as this may avoid potentially serious complications of urinary retention and its sequelae. Given the lack of data and guidelines, the decision as to whether to perform a male sling versus AUS depends on several factors including patient preference, surgeon comfort, and experience with the available procedures, and knowledge of the currently available outcomes and complications of each procedure.

Regardless of the treatment selected, it is important to review with patients appropriate immediate and long-term expectations following the procedure as well as potential complications and need for additional procedures.

## 7. Future Perspectives

The treatment of male SUI has evolved significantly over the past 40 years, with numerous improvements made to the AUS and multiple variations of the male sling developed in a relatively short period of time. And given the prevalence of prostate cancer with need for ongoing treatments, there will likely remain a significant need for treatment of iatrogenic SUI for the foreseeable future. It is anticipated that there will be ongoing improvements to the AUS to increase device longevity, reduce infectivity, and to better cater to patients with limited manual/mental capability. It is similarly expected that new variations and improvements to the existing male slings will continue to be developed, with further studies performed providing additional and longer-term followup on previously installed slings.

Novel techniques and materials will emerge to meet the ongoing need for alternative, minimally invasive, and adjustable options for management of low-to-moderate volume incontinence. This is particularly relevant in the setting of the recent FDA announcement in July of 2011 regarding utilization of mesh in pelvic organ prolapse. This may directly or indirectly impact the utilization of mesh material in other applications, including male sling placement.

More recently, investigators have examined the potential use of stem cell therapy to directly treat the underlying disease process. Several investigators have reported on the successful creation of muscle- or adipose-derived stem cells and hypothesized their potential use as a regenerative treatment for iatrogenic injury to the native rhabdosphincter [66–69]. Yamamoto and colleagues reported the use of adipose-derived stem cell injection into the periurethral tissues of two men with SUI following RRP performed at least two years prior [70]. With limited followup at 12 weeks, both men experienced significant improvements in measured pad weights over a 4-day period. Although this study has significant limitations including the lack of a control group treated with periurethral bulking injection with adipose tissue alone, findings such as these will likely lead to additional research on the potential for stem cell therapy.

Similar to stem cell therapy, additional investigations continue in identifying alternative bulking agents for use as a periurethral injectable material [71]. Watanabe and colleagues recently reported improved objective findings in rats treated with adipose-derived mesenchymal stromal cells compared to those treated with standard collagen injections [72]. Although these treatments remain in the early stages of research, they offer potential significant advantages over currently available surgical therapies given their minimally invasive approach without need for synthetic material/device implantations.

## 8. Conclusions

Iatrogenic male stress urinary incontinence remains a significant problem impacting a large number of patients with resultant impairment of quality of life. Patients presenting with SUI should undergo a thorough history and physical examination with additional studies obtained as indicated. Several therapies are currently available for the treatment of low-to-moderate volume incontinence including the AUS and several variations of male slings (BAS, RTS, ARS, and quadratic sling). Patients with large-volume incontinence are best managed with AUS when found to be an appropriate surgical candidate. Complications of sling/AUS placement include temporary retention, perineal pain, infections, erosions, de novo urinary symptoms, and device malfunction. Patients desiring surgical management of SUI should be counseled as to expected outcomes as well as potential complications.

## Figures and Tables

**Table 1:** Comparison of results of bone anchored sling placement in adult males with SUI.

Study	Pts (No.)	Mean/med f/u (mo)	Pre-op continence (mean/med)	Success def	Success (%)	Improved (%)	No improvement (%)	Complications (%)	Notes
Cespedes and Jacoby [19]	58	6			26 (45)	11 (19)	21 (36)	None	
Ullrich and Comiter [20]	36	25	≥3 ppd	0 ppd	24 (67)	9 (25)	3 (8)	None	Mean pads decreased from 4.6 to 0.64
Comiter [21]	48	48	≥3 ppd	0 ppd	31 (65)	10 (21)	7 (15)	Infection 1 (2), erosion 1 (2), perineal pain × 3 months 7 (16), 2 (4) screw dislodgement	Mean pads decreased from 4.6 to 1.0
Castle et al. [22]	38	18		≤1 ppd	15 (40)			Infection 3 (8), erosion 1 (3), majority with significant perineal pain × 3-4 mo	
Gallagher et al. [23]	31	15		≤1 ppd	18 (58)			Infection 2 (6), Sling removal 4 (13)	3 pts lost to f/u, 4 pts w/sling removal
Fischer et al. [24]	62	15	Pad wt 352 g ± 43	PGI-I 1 question	36 (58)	6 (10)	26 (42)	Infection 3 (5), erosion 1 (2), pain >5 months 5 (8), total 13 (21)	Prospective study; 71% chance of success if preop pad weight <423 g
Guimarães et al. [25]	62	28		0 ppd	40 (65)	14 (23)	8 (13)	Infection 2 (3), pain 12 (19), bone anchor dislodgement 1 (2)	3 year f/u w/21/30 (70), 4 year 8/12 (67) successful
Giberti et al. [26]	40	35		pad weight 0-1 g	22 (55)	5 (13)	13 (33)	Infection 6 (15), de novo detrusor overactivity 2 (5), pain 29 (73)	
Athanasopoulos et al. [27]	43	24	See notes	≤1 ppd	22 (51)	8 (19)	13 (30)	Infection 5 (12), ne novo urgency 6 (14), pain 1 (2)	Pre-op incontinence: 1-2 pads in 6/43 (14), 3–5 pads in 23/43 (53), and ≥6 pads in 14/43 (33)
Carmel et al. [28]	45	36		0 ppd	16 (36)	18 (40)	11 (24)	Infection 1 (2), pain ≤ 3 months 10 (22)	12 pts (27%) w/preop radiation

**Table 2:** Comparison of results of retrourethral transobturator sling placement in adult males with SUI.

Study	Pts (No.)	Mean/med f/u (mo)	Pre-op continence (mean/med)	Success def	Success (%)	Improved (%)	No improvement (%)	Complications (%)	Notes
Rehder et al. [33]	118	12	2.3 ppd	≤1 ppd	87 (74)	20 (17)	11 (9)	Retention 6 (5), perineal pain 23 (20)	Prospective
Cornel et al. [34]	35	12		0 ppd, <2 g urine loss/day	3 (9)	16 (46)	16 (46)	Removal 1 (3), retention 1 (3), mod-severe pain <3 months 12 (34)	Prospective, 2-center study
*Christine and Knoll [35]	19	13		0 ppd	15 (79)	4 (21)	0 (0)	None	Pts w/recurrent incontinence after AUS placement
*Soljanik et al. [36]	29	17	4.3 ppd	0 ppd	10 (35)	16 (55)	3 (10)	Urethral injury 1 (3), retention <2 wks 9 (24)	Pts previously failed sling placement
*Bauer et al. [37]	24	18	4.5 ppd	≤1 ppd	6 (25)	6 (25)	12 (50)	Removal 1 (4)	All patients with RP and adjuvant radiotherapy
Cornu et al. [38]	136	21	2.1 ppd	0 ppd	84 (62)	22 (16)	30 (22)	Pain 14 (10), dysuria 19 (14)	Prospective study; failure ass'd w/24-hr pad >200 g, trend toward radiation therapy
Bauer et al. [37]	126	27	4.9 ppd	≤1 ppd	65 (52)	30 (24)	31 (25)	Removal 2 (2), retention <10 wks 19 (15), persistent pain 1 (1)	Prospective study; 17 pts (14%) with pre-op radiation
Berger et al. [39]	26	22	5.6 ppd	0 ppd	16 (62)	7 (27)	3 (12)	Pain ≤4 weeks 5 (19)	

*Salvage patient populations.

**Table 3:** Comparison of results of adjustable retropubic sling placement in adult males with SUI.

Study	Pts (No.)	Mean/med f/u (mo)	Pre-op continence (mean/med)	Success def	Success (%)	Improved (%)	No improvement (%)	Complications (%)	Notes
Romano et al. [45]	48	45		0 ppd	31 (66)	6 (13)	10 (21)	Infection 3 (6), erosion 6 (13), removal 9 (19), pain 2 (4)	Argus sling; adjustment required in 5 (10)
*Tuygun et al. [46]	8	10	6.8 ppd	0 ppd	1 (13)	1 (13)	6 (75)	Pain 3 (38)	Argus sling; pts w/prior AUS erosion; study compares AUS to sling as salvage option
Bochove-Overgaauw and Schrier [47]	95	27	See notes	≤1 ppd	51 (54)	17 (18)	27 (28)	Infection 6 (6), erosion 3 (3), removal 11 (12), total 55 (58)	Argus sling; Pre-op continence: 1-2 ppd in 13/100, 3–5 ppd in 46/100, and 6–10 ppd in 41/100
Dalpiaz et al. [48]	29	35	5 ppd		5 (17)			Infection 2 (7), erosion 3 (10), bladder perforation 3 (10), retention 10 (35), removal 10 (35), significant pain 8 (27)	Argus sling; Dissatisfied with clinical outcome 21 (72)
Hübner et al. [49]	101	25		≤1 g 20-min pad test	80 (79)			Bladder perforation 5 (5), infection 5 (5), erosion 13 (13), removal 16 (16), pain <3 wks 15 (15)	Argus sling; adjustment required in 39 (39)
Sousa-Escandón et al. [50]	51	32		≤1 ppd	33 (65)	10 (20)	8 (16)	Infection 3 (6), removal 1 (2), bladder perforation 5 (10), majority with perineal pain	Remeex sling; multicenter study; adjustment required in 51 (100)
Verdejo et al. [51]	5	15	5–8 ppd	≤1 ppd	5 (100)				Remeex sling
Parra et al. [52]	15	19			5/12 (42)	4/12 (33)	3/12 (25)	Removal 3 (21), readjustment 12 (86), bladder perforation 4 (29), retention 5 (36)	Remeex sling

PPD: pads per day; PGI-I: Patient Global Impression of Improvement; RP: radical prostatectomy; RLPP: retrograde leak point pressure.

*Represents specialized/salvage patient populations.

**Table 4:** Comparison of results of single-cuff AUS placement in adult males with SUI.

Study	Pts (No.)	Continence def (pads/day)	Mean follow-up (yr)	Success (%)	Explantation (%)	Complications	(%)
	58	≤2	4.2	91.4	20.3	Mechanical failure	6.5
Arai et al. [53]						Infection	14
						Erosion	4.7

	124	0-1	6.8	82	36 (incl revision)	Mechanical failure	29
Kim et al. [54]						Infection	7
						Erosion	10

	25	0-1	6.2	61	16	Mechanical failure	0
O'Connor et al. [55]						Infection	8
						Erosion	8
						Atrophy	4

	218	0-1	3	69	27.1 (incl revision)	Mechanical failure	6
Lai et al. [56]						Infection	5.5
						Erosion	6
						Atrophy	9.6

	71	0-1	7.7	59	29 (incl revision)	Mechanical failure	25
Gousse et al. [57]						Infection	1.4
						Erosion	4

## Abbreviations

ARS: Adjustable retropubic sling
AUS: Artificial urinary sphincter
BAS: Bone-anchored sling
PPD: Pad(s) per day
PPI: Postprostatectomy incontinence
SUI: Stress urinary incontinence
RP: Radical prostatectomy
RTS: Retrourethral transobturator sling
TURP: Transurethral resection of the prostate.

## References

[1] Markland AD, Richter HE, Fwu C-W, Eggers P, Kusek JW (2011). Prevalence and trends of urinary incontinence in adults in the United States, 2001 to 2008. *Journal of Urology*.

[2] Anger JT, Saigal CS, Stothers L, Thom DH, Rodríguez LV, Litwin MS (2006). The prevalence of urinary incontinence among community dwelling men: results from the National Health and Nutrition Examination survey. *Journal of Urology*.

[3] Abrams P, Cardozo L, Fall M (2002). The standardisation of terminology of lower urinary tract function: report from the standardisation sub-committee of the international continence society. *Neurourology and Urodynamics*.

[4] Walsh PC, Marschke P, Ricker D, Burnett AL (2000). Patient-reported urinary continence and sexual function after anatomic radical prostatectomy. *Urology*.

[5] Gray M, Petroni GR, Theodorescu D (1999). Urinary function after radical prostatectomy: a comparison of the retropubic and perineal approaches. *Urology*.

[6] Lepor H, Kaci L, Xue X (2004). Continence following radical retropubic prostatectomy using self-reporting instruments. *Journal of Urology*.

[7] Eden CG, Arora A, Hutton A (2011). Cancer control, continence, and potency after laparoscopic radical prostatectomy beyond the learning and discovery curves. *Journal of Endourology*.

[8] Treiyer A, Anheuser P, Btow Z, Steffens J (2011). A single center prospective study: prediction of postoperative general quality of life, potency and continence after radical retropubic prostatectomy. *Journal of Urology*.

[9] Burkhard FC, Kessler TM, Fleischmann A, Thalmann GN, Schumacher M, Studer UE (2006). Nerve sparing open radical retropubic prostatectomy—does it have an impact on urinary continence?. *Journal of Urology*.

[10] Wasson JH, Reda DJ, Bruskewitz RC, Elinson J, Keller AM, Henderson WG (1995). A comparison of transurethral surgery with watchful waiting for moderate symptoms of benign prostatic hyperplasia. *New England Journal of Medicine*.

[11] Scalliet PGM, Remouchamps V, Curran D (2004). Retrospective analysis of results of p(65)+Be neutron therapy for treatment of prostate adenocarcinoma at the cyclotron of Louvain-la-Leuve. Part II: side effects and their influence on quality of life measured with QLQ-C30 of EORTC. *International Journal of Radiation Oncology Biology Physics*.

[12] Shipley WU, Zietman AL, Hanks GE (1994). Treatment related sequelae following external beam radiation for prostate cancer: a review with an update in patients with stages T1 and T2 tumor. *Journal of Urology*.

[13] Scott FB (1978). The artificial sphincter in the management of incontinence in the male. *Urologic Clinics of North America*.

[14] Avery K, Donovan J, Peters TJ, Shaw C, Gotoh M, Abrams P (2004). ICIQ: a brief and robust measure for evaluating the symptoms and impact of urinary incontinence. *Neurourology and Urodynamics*.

[15] Litwin MS, Lubeck DP, Henning JM, Carroll PR (1998). Differences in urologist and patient assessments of health related quality of life in men with prostate cancer: results of the capsure database. *Journal of Urology*.

[16] Abrams P, Andersson KE, Birder L (2010). Fourth international consultation on incontinence recommendations of the international scientific committee: evaluation and treatment of urinary incontinence, pelvic organ prolapse, and fecal incontinence. *Neurourology and Urodynamics*.

[17] Lai HH, Hsu EI, Boone TB (2009). Urodynamic testing in evaluation of postradical prostatectomy incontinence before artificial urinary sphincter implantation. *Urology*.

[18] Dikranian AH, Chang JH, Rhee EY, Aboseif SR (2004). The male perineal sling: comparison of sling materials. *Journal of Urology*.

[19] Cespedes RD, Jacoby K (2001). Male slings for postprostatectomy incontinence. *Techniques in Urology*.

[20] Ullrich NF, Comiter CV (2004). The male sling for stress urinary incontinence: 24-month followup with questionnaire based assessment. *The Journal of urology*.

[21] Comiter CV (2005). The male perineal sling: intermediate-term results. *Neurourology and Urodynamics*.

[22] Castle EP, Andrews PE, Itano N, Novicki DE, Swanson SK, Ferrigni RG (2005). The male sling for post-prostatectomy incontinence: mean followup of 18 months. *Journal of Urology*.

[23] Gallagher BL, Dwyer NT, Gaynor-Krupnick DM, Latini JM, Kreder KJ (2007). Objective and quality-of-life outcomes with bone-anchored male bulbourethral sling. *Urology*.

[24] Fischer MC, Huckabay C, Nitti VW (2007). The male perineal sling: assessment and prediction of outcome. *Journal of Urology*.

[25] Guimarães M, Oliveira R, Pinto R (2009). Intermediate-term results, up to 4 years, of a bone-anchored male perineal sling for treating male stress urinary incontinence after prostate surgery. *BJU International*.

[26] Giberti C, Gallo F, Schenone M, Cortese P, Ninotta G (2009). The bone anchor suburethral synthetic sling for iatrogenic male incontinence: critical evaluation at a mean 3-year followup. *Journal of Urology*.

[27] Athanasopoulos A, Konstantinopoulos A, McGuire E (2010). Efficacy of the InVance male sling in treating stress urinary incontinence: a three-year experience from a single centre. *Urologia Internationalis*.

[28] Carmel M, Hage B, Hanna S, Schmutz G, Tu LM (2010). Long-term efficacy of the bone-anchored male sling for moderate and severe stress urinary incontinence. *BJU International*.

[29] Soljanik I, Gozzi C, Becker AJ, Stief CG, Bauer RM (2012). Risk factors of treatment failure after retrourethral transobturator male sling. *World Journal of Urology*.

[30] Render P, Von Gleissenthall GF, Pichler R, Glodny B (2009). The treatment of prostatectomy incontinence with retroluminal transobtulator repositioning sling advancer. Lessons learnt from accumulative experience. *Archivos Espanoles de Urologia*.

[31] Elzevier HW, Cornel EB (2010). The 1-year outcome of the transobturator retroluminal repositioning sling in the treatment of male stress urinary incontinence. *BJU International*.

[32] Rehder P, Gozzi C (2007). Transobturator sling suspension for male urinary incontinence including post-radical prostatectomy. *European Urology*.

[33] Rehder P, Mitterberger MJ, Pichler R, Kerschbaumer A, Glodny B (2010). The 1 year outcome of the transobturator retroluminal repositioning sling in the treatment of male stress urinary incontinence. *BJU International*.

[34] Cornel EB, Elzevier HW, Putter H (2010). Can advance transobturator sling suspension cure male urinary postoperative stress incontinence?. *Journal of Urology*.

[35] Christine B, Knoll LD (2010). Treatment of recurrent urinary incontinence after artificial urinary sphincter placement using the advance male sling. *Urology*.

[36] Soljanik I, Becker AJ, Stief CG, Gozzi C, Bauer RM (2010). Repeat retrourethral transobturator sling in the management of recurrent postprostatectomy stress urinary incontinence after failed first male sling. *European Urology*.

[37] Bauer RM, Soljanik I, Füllhase C (2011). Results of the AdVance transobturator male sling after radical prostatectomy and adjuvant radiotherapy. *Urology*.

[38] Cornu J-N, Sèbe P, Ciofu C, Peyrat L, Cussenot O, Haab F (2011). Mid-term evaluation of the transobturator male sling for post-prostatectomy incontinence: focus on prognostic factors. *BJU International*.

[39] Berger AP, Strasak A, Seitz C, Rein P, Hobisch A (2011). Single institution experience with the transobturator sling suspension system advance in the treatment of male urinary incontinence: mid-term results. *International Brazilian Journal of Urology*.

[40] Elliott DS, Barrett DM, Gohma M, Boone TB (2001). Does nocturnal deactivation of the artificial urinary sphincter lessen the risk of urethral atrophy?. *Urology*.

[41] Brito CG, Mulcahy JJ, Mitchell ME, Adams MC (1993). Use of a double cuff AMS800 urinary sphincter for severe stress incontinence. *Journal of Urology*.

[42] DiMarco DS, Elliott DS (2003). Tandem cuff artificial urinary sphincter as a salvage procedure following failed primary sphincter placement for the treatment of post-prostatectomy incontinence. *Journal of Urology*.

[43] Guralnick ML, Miller E, Toh KL, Webster GD (2002). Transcorporal artificial urinary sphincter cuff placement in cases requiring revision for erosion and urethral atrophy. *Journal of Urology*.

[44] Aaronson DS, Elliott SP, McAninch JW (2008). Transcorporal artificial urinary sphincter placement for incontinence in high-risk patients after treatment of prostate cancer. *Urology*.

[45] Romano SV, Metrebian SE, Vaz F (2009). Long term results of a phase III multicentre trial of the adjustable male sling for treating urinary incontinence after prostatectomy: minimum 3 years. *Actas Urologicas Espanolas*.

[46] Tuygun C, Imamoglu A, Gucuk A, Goktug G, Demirel F (2009). Comparison of outcomes for adjustable bulbourethral male sling and artificial urinary sphincter after previous artificial urinary sphincter erosion. *Urology*.

[47] Bochove-Overgaauw DM, Schrier BPh (2011). An adjustable sling for the treatment of all degrees of male stress urinary incontinence: retrospective evaluation of efficacy and complications after a minimal followup of 14 months. *Journal of Urology*.

[48] Dalpiaz O, Knopf HJ, Orth S, Griese K, Aboulsorour S, Truss M (2011). Mid-term complications after placement of the male adjustable suburethral sling: a single center experience. *Journal of Urology*.

[49] Hübner WA, Gallistl H, Rutkowski M, Huber ER (2011). Adjustable bulbourethral male sling: experience after 101 cases of moderate-to-severe male stress urinary incontinence. *BJU International*.

[50] Sousa-Escandón A, Cabrera J, Mantovani F (2007). Adjustable suburethral sling (male remeex system) in the treatment of male stress urinary incontinence: a multicentric European study. *European Urology*.

[51] Verdejo PN, Costa YP, Domínguez FO (2010). Our experience in the treatment of male stress urinary incontinence with the male remeex system. *Archivos Espanoles de Urologia*.

[52] Parra JDJ, Lostal JLC, Alfaro AH (2010). REMEEX system for the treatment of male urinary stress incontinence: our experience. *Actas Urologicas Espanolas*.

[53] Arai Y, Takei M, Nonomura K (2009). Current use of the artificial urinary sphincter and its long-term durability: a nationwide survey in Japan. *International Journal of Urology*.

[54] Kim SP, Sarmast Z, Daignault S, Faerber GJ, McGuire EJ, Latini JM (2008). Long-term durability and functional outcomes among patients with artificial urinary sphincters: a 10-year retrospective review from the University of Michigan. *Journal of Urology*.

[55] O’Connor RC, Lyon MB, Guralnick ML, Bales GT (2008). Long-term follow-up of single versus double cuff artificial urinary sphincter insertion for the treatment of severe postprostatectomy stress urinary incontinence. *Urology*.

[56] Lai HH, Hsu EI, Teh BS, Butler EB, Boone TB (2007). 13 years of experience with artificial urinary sphincter implantation at Baylor College of Medicine. *Journal of Urology*.

[57] Gousse AE, Madjar S, Lambert MM, Fishman IJ (2001). Artificial urinary sphincter for post-radical prostatectomy urinary incontinence: long-term subjective results. *Journal of Urology*.

[58] Bauer RM, Soljanik I, Füllhase C (2011). Mid-term results for the retroluminar transobturator sling suspension for stress urinary incontinence after prostatectomy. *BJU International*.

[59] Comiter CV, Nitti V, Elliot C, Rhee E (2012). A new quadratic sling for male stress incontinence: retrograde leak point pressure as a measure of urethral resistance. *Journal of Urology*.

[60] Saffarian A, Walsh K, Walsh IK, Stone AR (2003). Urethral atrophy after artificial urinary sphincter placement: is cuff downsizing effective?. *Journal of Urology*.

[61] Rahman NU, Minor TX, Deng D, Lue TF (2005). Combined external urethral bulking and artificial urinary sphincter for urethral atrophy and stress urinary incontinence. *BJU International*.

[62] Trost L, Elliott D (2012). Small intestinal submucosa urethral wrap at the time of artificial urinary sphincter placement as a salvage treatment option for patients with persistent/recurrent incontinence following multiple prior sphincter failures and erosions. *Urology*.

[63] Kahlon B, Baverstock RJ, Carlson KV (2011). Quality of life and patient satisfaction after artificial urinary sphincter. *Journal of the Canadian Urological Association*.

[64] Magera JS, Elliott DS (2008). Artificial urinary sphincter infection: causative organisms in a contemporary series. *Journal of Urology*.

[65] Venn SN, Greenwell TJ, Mundy AR (2000). The long-term outcome of artificial urinary sphincters. *Journal of Urology*.

[66] Smaldone MC, Chancellor MB (2008). Muscle derived stem cell therapy for stress urinary incontinence. *World Journal of Urology*.

[67] Smaldone MC, Chen ML, Chancellor MB (2009). Stem cell therapy for urethral sphincter regeneration. *Minerva Urologica e Nefrologica*.

[68] Sumino Y, Hirata Y, Hanada M, Akita Y, Sato F, Mimata H (2011). Long-term cryopreservation of pyramidalis muscle specimens as a source of striated muscle stem cells for treatment of post-prostatectomy stress urinary incontinence. *Prostate*.

[69] Lin G, Wang G, Banie L (2010). Treatment of stress urinary incontinence with adipose tissue-derived stem cells. *Cytotherapy*.

[70] Yamamoto T, Gotoh M, Hattori R (2010). Periurethral injection of autologous adipose-derived stem cells for the treatment of stress urinary incontinence in patients undergoing radical prostatectomy: report of two initial cases. *International Journal of Urology*.

[71] Kwon D, Kim Y, Pruchnic R (2006). Periurethral cellular injection: comparison of muscle-derived progenitor cells and fibroblasts with regard to efficacy and tissue contractility in an animal model of stress urinary incontinence. *Urology*.

[72] Watanabe T, Maruyama S, Yamamoto T (2011). Increased urethral resistance by periurethral injection of low serum cultured adipose-derived mesenchymal stromal cells in rats. *International Journal of Urology*.

